# Complete genome sequence of *Microbacterium* sp. PI-SA-G, a polyextremotolerant bacterium isolated from *Festuca orthophylla* in the Salar de Aguilar (Atacama Desert–Chile)

**DOI:** 10.1128/mra.00555-26

**Published:** 2026-06-29

**Authors:** Juan Castro-Severyn, Safka Hernandez, Bastián León, Enzo Galliani, Matías Vargas-Reyes, Denisse Bravo, José Manuel Pérez-Donoso

**Affiliations:** 1 Pewman Innovation SpA, Santiago, Chile; 2 Facultad de Odontología, Universidad Andres Bello 28087, Santiago, Chile; 3 Center for Bioinformatics, and Integrative Biology (CBIB), Facultad de Ciencias Biológicas, Universidad Andres Bello 28087, Santiago, Chile; Montana State University, Bozeman, Montana, USA

**Keywords:** *Microbacterium*, extremotolerant, photoprotection, atacama desert, plant associated

## Abstract

*Microbacterium* sp. PI-SA-G is a pigmented and extremotolerant bacterium isolated from *Festuca orthophylla* plant aerial tissue in the high-altitude Salar Aguilar (Atacama Desert). Its complete and single circular chromosome (3.72 Mb) encodes multiple stress-tolerance and plant growth-promoting traits. Also, average nucleotide identity (ANI) (84.5%) and digital DNA-DNA hybridization (dDDH) (37%) values fall below established species thresholds.

## ANNOUNCEMENT

*Microbacterium* is a gram-positive, aerobic, non-spore-forming actinobacterial genus described originally by Orla-Jensen (1) and subsequently emended by Takeuchi and Hatano (2). The genus accounts for more than 90 species isolated from a wide range of habitats and hosts (3), reflecting a cosmopolitan distribution spanning soil, plants, freshwater, marine sediments, sludges, clinical, and extreme environments (4).

Here we report the genome sequence of *Microbacterium* sp. strain PI-SA-G, providing a genomic resource for studying plant-microbe interactions in extreme Atacama environments. The strain was isolated from leaves of *Festuca orthophylla* (Poaceae) collected during a 2025 expedition to Salar de Aguilar (25°51′07.7″S, 68°55′28.9″W). This endorheic basin (3,800 masl; 589 km²) in the Atacama Region features extreme conditions [scarce rainfall, high evaporation, low average temperature (2°C)] (5). For isolation, 5 g of plant tissue were suspended in 20 mL of Tris buffer (20 mM, pH 7.4), shaken for 1 h, and 100 µL were plated on R2A agar at 28°C. A yellow-pigmented colony (PI-SA-G) was selected and cultured in LB (28°C, 180 rpm, 24 h); cells were pelleted (18,000 × *g* , 10 min), and total DNA was extracted (Quick-DNA Fecal/Soil Microbe kit, Zymo). The sequencing library was constructed with the Rapid Barcoding Kit 24 V14 (Oxford Nanopore Technologies) without shearing or size selection using 200 ng of DNA. The library was loaded on a FLO-MIN114 (R10) Flow Cell and sequenced on a MinION platform (Oxford Nanopore Technologies), controlled via MinKNOW v25.03.9 with Dorado v0.9.2 as basecaller (all tools were run with default parameters unless otherwise specified). Output reads quality control was performed using NanoPlot v1.46.2 for evaluation, Porechop v0.2.4 for adapter removal and NanoFilt v2.8.0 for filtering and trimming [*N*_s_ = 0, length ≥ 500 bp and *Q* ≥ 20 (6)]. Assembly was performed using Flye v2.9.2 (7), which identifies and resolves the circular overlaps; the assembly was polished with Medaka v2.2.1 and quality metrics were assessed using QUAST v5.3.0 (8) (Table 1). Genome completeness and contamination were evaluated using CheckM2 v1.1.0 (9), and taxonomic assignment using GTDB-Tk v2.7.0 (10). Genome annotation was carried out with NCBI PGAP v6.11 (11) and genome visualization was generated using Proksee (12). A phylogeny for the PI-SA-G strain (including 10 reference genomes) was inferred using FastTree v2.2.0 (13) based on an alignment of single-copy core genes generated with PIRATE v1.0.5 (14) and the tree was visualized using FigTree v1.4.4.

**TABLE 1:** Genome features of PI-SA-G strain

Parameter	Value
Post filter reads (#)	155,547
Read N50 (bp)	6,121
Genome size (bp)	3,727,191
Contigs (#)	1
N50 (bp)	3,727,191
L50 (#)	1
Genome Coverage (X)	123
Completion (%)	99.67
Contamination (%)	0.86
GC Content (%)	68.57
Coding region (%)	92.45
Intergenic region (%)	7.55
Genes (#)	3,685
Proteins (#)	3,599
Hypothetical Proteins (#)	478
Pseudo genes (#)	31
rRNAs (#)	6 (5S: 2; 16S: 2; 23S: 2)
tRNAs (#)	46
ncRNAs (#)	3

**Fig 1:**
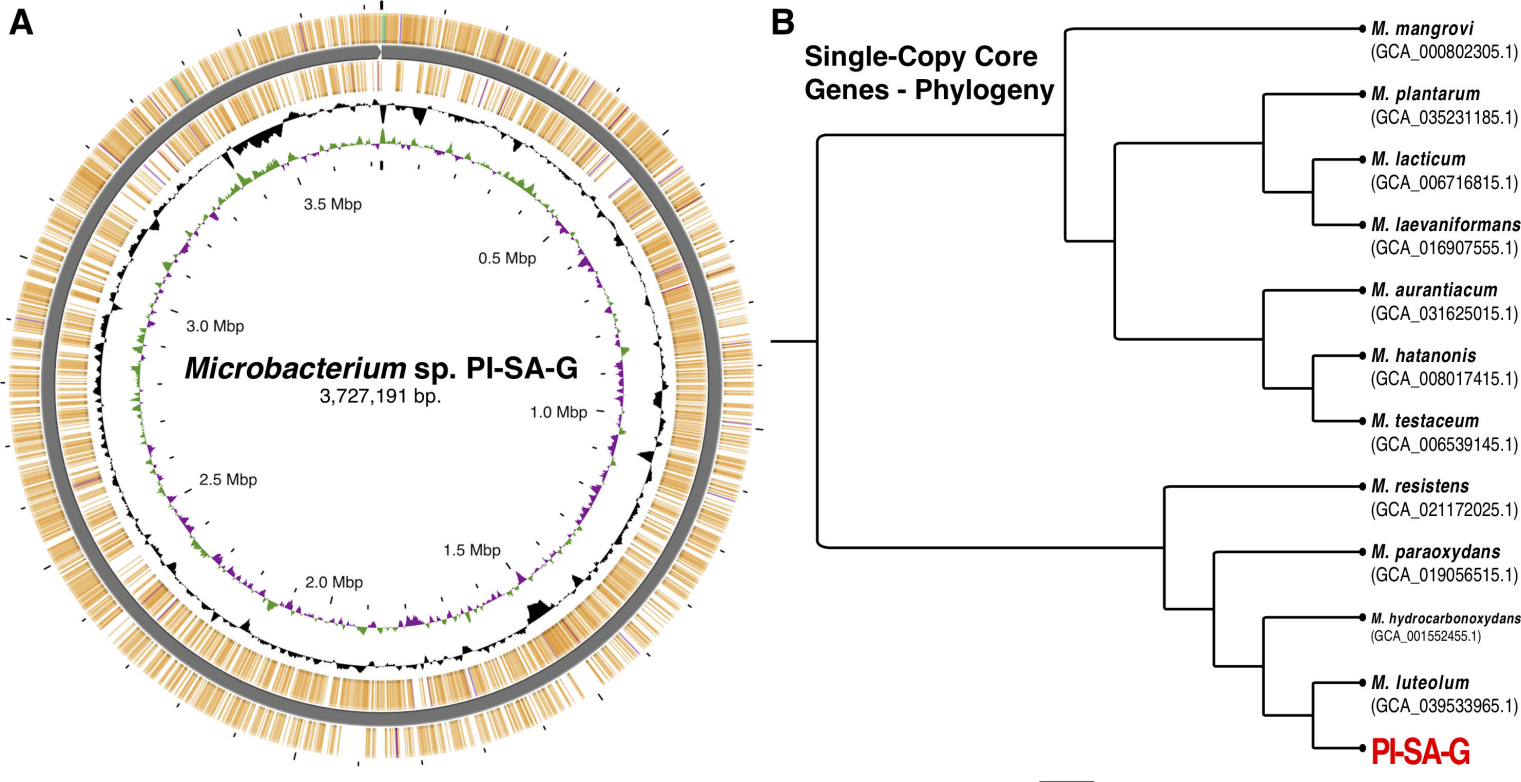
Genome overview and phylogenetic placement of *Microbacterium* sp. PI-SA-G. (**A**) Circular representation of the PI-SA-G genome. The map displays genomic features including genome size (3,727,191 bp) and structural organization. Concentric rings represent coding sequences on forward and reverse strands, chromosome, and additional genomic features (GC content and GC skew). (**B**) Phylogenetic tree inferred from the alignment of single-copy core genes, showing the evolutionary relationship between *Microbacterium* sp. PI-SA-G and reference genomes from *Microbacterium* species. The tree was constructed using a concatenated alignment of core genes, and branch lengths indicate the number of substitutions per site. Scale bar represents evolutionary distance.

The complete genome of strain PI-SA-G consists of a single circular chromosome (confirmed by the Flye generated assembly graph; 3.72 Mb) with high guanine cytosine (GC) content (68.6%) and 92.45% coding density (Fig. 1A). Genome-based taxonomy assigned it to the genus *Microbacterium*, but ANI (84.5% vs. *M. luteolum* JCM 9174) and dDDH (<37%) values suggest a novel species. Core gene phylogeny places PI-SA-G close to *M. luteolum*, forming a distinct branch related to *M. resistens* (Fig. 1B). The genome encodes plant-associated traits (indole-3-acetic acid [IAA] precursors, siderophore transport, phosphatases, vitamin pathways, and multiple B12 transporters) and extremotolerance features, including UV protection (*crtN*, *phrA*, *uvrA*), oxidative stress defenses, and metal resistance (As, Cu, Ni), reflecting adaptation to Atacama Desert conditions.

## Data Availability

The genome sequence of *Microbacterium sp*. PI-SA-G has been deposited in DDBJ/ENA/GenBank under accession JBXVTS000000000 and the raw data under accession SRR38387354 (BioProject PRJNA1457443).
